# Depression, Diabetes and Dementia: Formaldehyde May Be a Common Causal Agent; Could Carnosine, a Pluripotent Peptide, Be Protective?

**DOI:** 10.14336/AD.2017.0120

**Published:** 2017-04-01

**Authors:** Alan R. Hipkiss

**Affiliations:** Aston Research Centre for Healthy Ageing (ARCHA), Aston University, Birmingham, B4 7ET, U.K

Recent studies have highlighted the possible involvement of formaldehyde in a number of age-associated phenomena, dementia, depression and diabetes (type-2). Papers by Li et al. [1,2], Cui et al. [3], Tong et al. [4,5] and Mei et al. [6] have shown an association between formaldehyde and age-related dysfunction such as glaucoma, stroke, Alzheimer’s disease and memory decline. Furthermore, Tulpule and colleagues [7,8] have demonstrated that formaldehyde strongly stimulates glycolytic flux in astrocytes and neurones, implying its contribution to metabolic age-related dysfunction, (e.g. type-2 diabetes, memory loss and neurodegeneration). Formaldehyde is thought to induce macromolecular dysfunction, at least in part, via its ability to crosslink protein to DNA [9].

Although formaldehyde-induced toxicity is not an entirely a new idea, it has been suggested that the presence of formaldehyde in the brain has been over-looked as either causative or a consequence of other underlying and deleterious phenomena [10,11]. Furthermore, given that current research indicates that excessive glycolytic activity may be an important contributor to not only type-2 diabetes but also to neurodegenerative conditions, it is perhaps relevant to note that formate, a product of formaldehyde metabolism, has been shown to enhance glycolytic flux in cultured astrocytes and neurones but inhibit mitochondrial respiration [7,8]. Other studies have shown that formaldehyde’s effects on norepinephrine are mediated by direct reaction with the hormone, thus decreasing hormone availability [6] and thereby contributing to age-related memory decline.

It has been found that the carbonyl scavengers, aminoguanidine and resveratrol, are effective in suppressing the effects of formaldehyde [12], but due to its toxicity, aminoguanidine is unlikely to be employed therapeutically, and resveratrol has also been described as a formaldehyde donor [13]. The naturally-occurring, pluripotent dipeptide, carnosine can also suppress the cross-linking activity of formaldehyde [14], most probably by reacting directly with it. More recently, carnosine has been shown to be protective in rats exposed to formaldehyde [15]. It is also interesting to note that increased levels of endogenous formaldehyde are present in the brains of the senescence accelerated mouse strain, SAMP8, at 3 months of age [16]. In earlier studies, dietary supplementation with carnosine has been shown to delay the onset of age-related changes in another senescence accelerated mouse strain (SAMP1) as well as suppress brain mitochondrial monoamine oxidase activity [17]. Thus, carnosine’s beneficial effects on the SAMP1 mice might be explained, at least in part, not only by the dipeptide’s reactivity towards formaldehyde, but also by suppressing amine oxidase-mediated formaldehyde generation from methylamine.

Carnosine has also been shown to suppress glycolysis [18,19] but stimulate mitochondrial activity in various model cell systems [20-23] and delay cell senescence in cultured human fibroblasts [24]. Given carnosine’s well-characterized ability to scavenge a variety of deleterious aldehydes, including methylglyoxal (MG) [25,26] the toxic by-product of excessive glycolysis responsible for much macromolecular modification associated with the secondary complications of type-2 diabetes, it is suggested that carnosine, a relatively non-toxic dipeptide, could be explored for its protective activity against reactive carbonyl compounds generally and formaldehyde in particular. Additionally, carnosine’s ability to inhibit regional brain monoamine-oxidase activity [27] could contribute to its antidepressant actions [28] by maintaining norepinephrine levels [29] by suppressing formaldehyde generation [30].

That Alzheimer’s disease, depression and type-2 diabetes may exhibit common metabolic features associated with increased formaldehyde generation, and which carnosine could ameliorate, directly or indirectly, suggests that its efficacy should be explored with respect to these age-related conditions. Administration via a nasal route could be a useful method to escape the effects of serum carnosinase, especially as the olfactory lobe is normally enriched in carnosine and loss of a sense of smell is frequently an early symptom of neurodegeneration. However, dietary supplementation with carnosine, especially if the related peptide anserine is also present, has been shown to improve aspects of brain function (behaviour, cognition and well-being) in a number of double-blind, placebo-controlled, human studies [31-36], which begins to question whether serum carnosinase is a major impediment to the dipeptide’s potential efficacy.

In summary, it has been shown that raised tissue levels of formaldehyde are associated with depression, neurodegeneration and type-2 diabetes. It is suggested that administration of the naturally-occurring dipeptide carnosine may exert alleviative effects on depression and memory by helping to maintain norepinephrine levels due to (i) inhibiting formaldehyde synthesis from methylamine via its action on monoamine oxidase, and (ii) scavenging formaldehyde, thereby decreasing norepinephrine inactivation. Both type-2 diabetes and Alzheimer’s disease are associated with enhanced protein glycation mediated mostly by MG, a glycolytic by-product. Carnosine’s formaldehyde scavenging activity will decrease formate synthesis, which would otherwise stimulate glycolysis, while the dipeptide’s suppressive effects on glycolysis together with its ability to scavenge MG, will decrease the potential for MG-mediated protein modification which characterises age-related protein dysfunction in type-2 diabetes and Alzheimer’s disease. (See Table 1 for concise summary). It is therefore suggested that various forms of carnosine therapy should be explored [37].

**Table 1:** Summary of the putative beneficial effects of carnosine on formaldehyde-mediated changes associated with depression, Alzheimer’s disease and type-2 diabetes.

Condition	Characteristic/possible protective mechanism	Refs.
*Depression*		
No carnosine	Increased HCHO and norepinephrine inactivation	[2,6,9]
Plus carnosine	HCHO scavenging.	[14]
	Decreased HCHO formation by inhibition of SSAO	
	mediated methylamine oxidation thus raising	
	norepinephrine activity	[17]
*AD & T2D*		
No carnosine	Increased HCHO and increased glycolysis	[3,4]
	Increased MG-mediated protein glycation	[5,7]
	Mitochondrial dysfunction	[10,11]
Plus carnosine	Decreased glycolysis and decreased protein glycation	
	due to decreased formate synthesis	[15,17]
	and direct inhibitory effects on glycolysis.	[18,19]
	MG and HCHO scavenging decreases glycation	[14,26]
	Increased mitochondrial activity	[20,21,23]

HCHO: formaldehyde. AD: Alzheimer’s disease. T2D: type-2 diabetes. MG: methylglyoxal
